# Pannexin1 knockout and blockade reduces ischemic stroke injury in female, but not in male mice

**DOI:** 10.18632/oncotarget.16937

**Published:** 2017-04-07

**Authors:** Moises Freitas-Andrade, John F. Bechberger, Brian A. MacVicar, Victor Viau, Christian C. Naus

**Affiliations:** ^1^ Department of Cellular and Physiological Sciences, The Life Science Institute, University of British Columbia, Vancouver, British Columbia, Canada; ^2^ Department of Psychiatry, Djavad Mowafaghian Centre for Brain Health, University of British Columbia, Vancouver, British Columbia, Canada

**Keywords:** stroke, neuroprotection, neuroinflammation, sex-differences, pannexin, Pathology Section

## Abstract

The membrane channel Pannexin 1 (Panx1) mediates apoptotic and inflammatory signaling cascades in injured neurons, responses previously shown to be sexually dimorphic under ischemic conditions. We tested the hypothesis that Panx1 plays an underlying role in mediating sex differences in stroke outcome responses. Middle-aged, 8-9 month old male and female wild type and Panx1 KO mice were subjected to permanent middle cerebral artery (MCA) occlusion, and infarct size and astrocyte and microglia activation were assessed 4 days later. The sexually dimorphic nature of Panx1 deletion was also explored by testing the effect of probenecid a known Panx1 blocker to alter stroke volume. Panx1 KO females displayed significantly smaller infarct volumes (~ 50 % reduction) compared to their wild-type counterparts, whereas no such KO effect occurred in males. This sex-specific effect of Panx1 KO was recapitulated by significant reductions in peri-infarct inflammation and astrocyte reactivity, as well as smaller infarct volumes in probenecid treated females, but not males. Finally, females showed overall, higher Panx1 protein levels than males under ischemic conditions. These findings unmask a deleterious role for Panx1 in response to permanent MCA occlusion, that is unique to females, and provide several new frameworks for understanding sex differences in stroke outcome.

## INTRODUCTION

Pannexin1 (Panx1) is a hexameric single membrane channel-forming protein with similar overall structure to connexin hemichannels; it is permeable to ions and small signalling molecules like ATP and glucose [1–3]. Among the three Panx family members (Panx1, Panx2 and Panx3), Panx1 is the most widely expressed [4] and it is linked to neuronal ischemic injury and inflammation induced by apoptotic cells [5–7]. Under ischemic conditions, in cortical and hippocampal neurons, Panx1 channels are activated resulting in irreversible current activation, cell swelling and membrane breakdown [7]. Moreover, overstimulation of NMDA receptors in ischemic hippocampal neurons promotes Panx1 channel opening by Src family kinases [8], resulting in a prolonged depolarizing inward current and cell death [8].

In addition to these anoxic depolarization properties, Panx1 has also been implicated in mediating inflammatory responses [5]. Cells undergoing apoptosis release chemotactic inflammatory factors to promote phagocytic removal of dead cells, including ATP and UTP, representing one class of ‘find-me’ signals released during the initial stages of cell death [9]. Panx1 contributes to this process, as it is induced by caspase activity in apoptotic cells to regulate ATP and UTP release [6, 9]. Panx1 has also been shown to associate with components of the multiprotein inflammasome complex, including the P2×7 receptor and caspase-1 [5]. The inflammasome is a key component of the innate immune response and is stimulated by cerebral ischemia [10].

While several lines of evidence linking Panx1 activity with inflammation [5], apoptosis [9], and necrosis [7] have been reported, studies investigating the effect of Panx1 in cerebral ischemia have rendered contradictory results. For instance, Bargiotas et al. (2011) demonstrated that genetic deletion of Panx1 did not significantly reduce neurodegeneration in mice subjected to permanent middle cerebral artery (MCA) occlusion [11]. In contrast, the use of probenecid, a drug known to affect organic anion transporters and shown to inhibit Panx1 [5], significantly reduced inflammation, cerebral edema and neuronal death, in male mice 48 hours after transient focal ischemia [12]. Similarly, Cisneros-Mejorado et al. (2015) using a transient MCA occlusion model reported significant infarct volume reduction in both male mice treated with Panx1 inhibitor Brillant Blue G and mefloquine (however see also [13]) as well as Panx1 KO animals. The authors suggested that Panx1 might be associated with deleterious signaling cascade leading to neuronal death [14]. However, Mahi et al. (2015) using mice of either sex reported that Panx1 is potentially neuroprotective in cerebral ischemia in their model of bilateral carotid artery occlusion (BCAO) followed by ischemic post-conditioning [15]. Taken together, the seemingly confounding results reported by different groups could be attributed by the different models used in these studies as well as the complex mechanisms associated with Panx1 and ischemia.

A possible contributing factor to the discrepancies found in the literature could be due to the sex (male or female) or perhaps the sex steroid hormone status of the animals, used in the studies. Stroke is one of the most strikingly sex-specific diseases in its epidemiology [16]. Despite both experimental and clinical evidence for sexual dimorphism in ischemic brain injury [16], the mechanisms, however, are not fully elucidated. Panx1 is associated with apoptosis as well as inflammation in injured neurons [5–7], and each of these responses are known to be sexually dimorphic in stroke [17–19]. Thus, in the present study we reasoned that male and female mice should show different responses to Panx1 KO after focal cerebral ischemia. Our findings indicate that the lack of Panx1 results in a marked reduction in infarct volume, inflammation and gliosis in female, but not in male mice. The same sex-specific effect in infarct volume was also observed by pharmacologically inhibiting Panx1 activity. This striking difference between males and females underscores Panx1 as an important focal point to explain sexual dimorphisms in stroke outcome.

## RESULTS

### Infarct volume responses to Panx1 deletion and blockade

**Figure 1:**
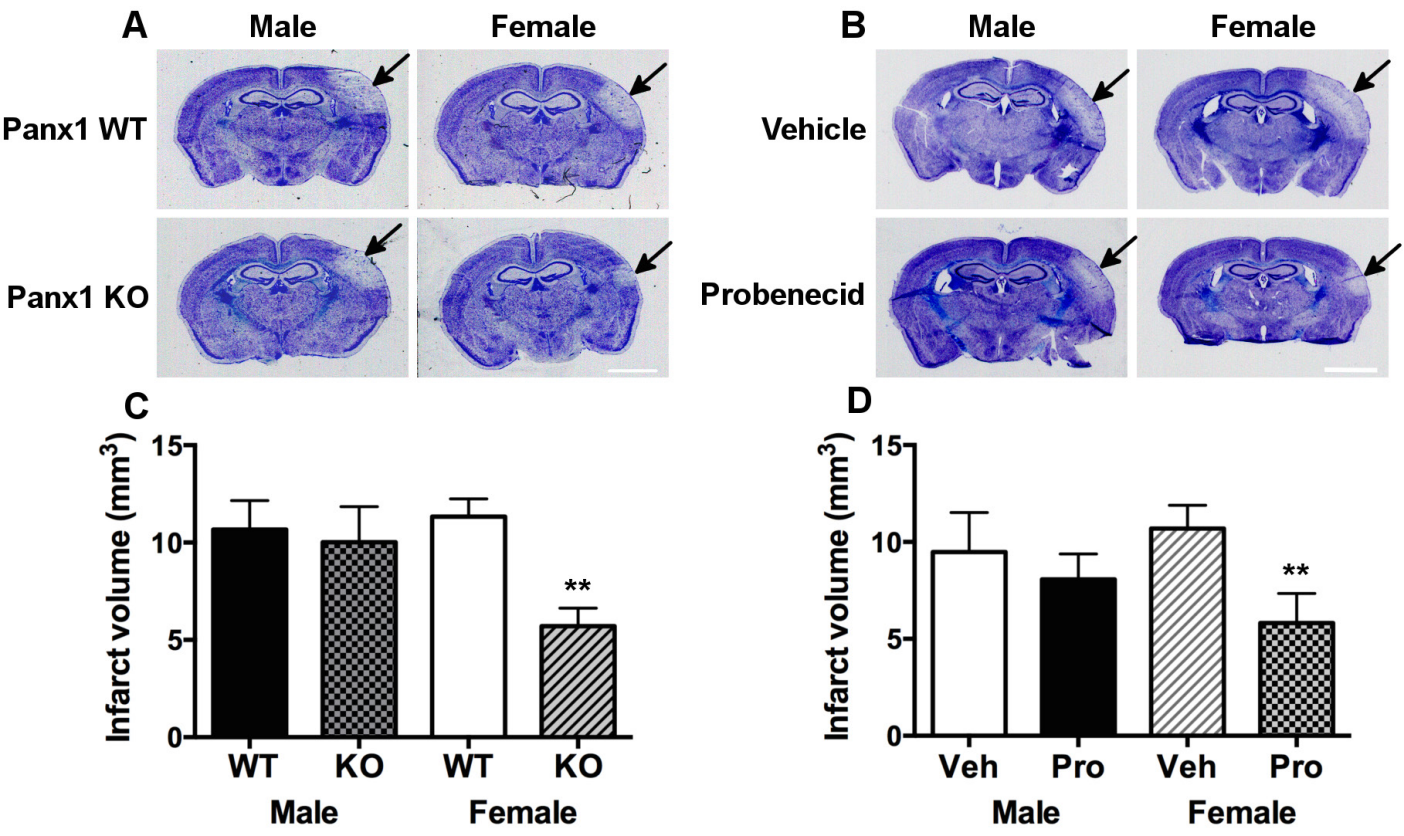
Panx1 KO and blockade is neuroprotective in female, but not in male mice Representative photomicrographs showing sex- and Panx1-dependent differences in infarct volume responses 4-days after permanent middle cerebral artery (MCA) occlusion in wild type and Panx1 knockout (KO) animals **A**, and in those treated with vehicle or the Panx1 blocker, probenecid **B**. Arrows point to region of infarct, scale bar = 2 mm in **A**. and **B**. Mean ± SEM infarct volumes as a function of Panx1 gene deletion in **C**. and Panx1 blockade in **D**. in male and females 4-days after permanent MCA occlusion. **P < 0.01 *vs* wildtype or vehicle treated, female counterpart; *n* = 7-9 and *n* = 6 animals per group in **C**. and **D**., respectively.

In order to determine whether Panx1 is linked to sex-specific mechanism(s) under ischemic conditions, both male and female WT and Panx1 KO mice were subjected to permanent MCA occlusion. Two-way analysis indicated a significant main effect of Panx1 genotype [F(1, 26) = 5.02; *P* = 0.034], no main effect of sex [F(1, 26) = 2.36; *P* = 0.136] and no significant interaction between sex and genotype [F(1, 26) = 3.18; *P* = 0.086]. Confirmed by post-hoc analysis, the effect of Panx1 deletion to decrease infarct volume was observed in female (*P* = 0.008), but not in male (*P* > 0.5) subjects (Figure 1A and 1C). With respect to Panx1 blockade, two-way analysis indicated a significant main effect of drug treatment [F(1, 20) = 5.62; *P* = 0.028], no main effect of sex [F(1, 20) = 0.42; *P* > 0.05] and no significant interaction between sex and treatment [F(1, 20) = 2.09; *P* = 0.163]. Confirmed by post-hoc analysis, the effect of Panx1 blockade to decrease infarct volume responses was observed in female (*P* = 0.014), but not in male (*P* > 0.5) subjects (Figure 1B and 1D).

### Neuroinflammatory responses to Panx1 deletion

**Figure 2:**
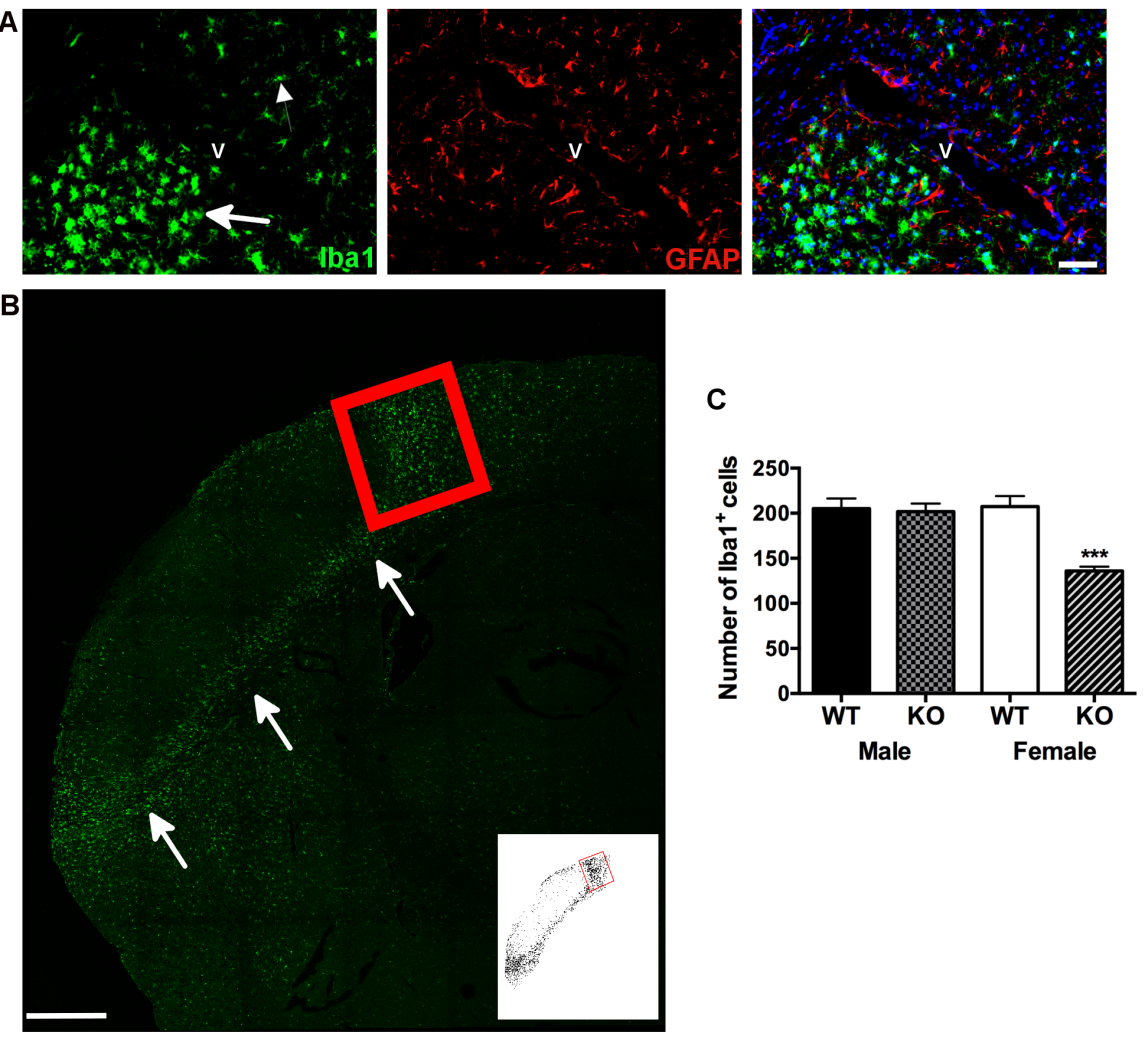
Neuroinflammation is reduced in female, but not in male Panx1 KO mice **A**. Immunofluorescent photomicrographs **A**. to illustrate microglia (Iba-1 positive, green) and astrocyte (GFAP positive, red) staining (left and middle panels, respectively), and concurrent labeling for these markers within identified (DAPI, blue) cell nuclei (right panel). Arrows indicate reactive microglia adjacent to the infarct. Smaller arrow indicates non-reactive microglia on the other side of blood vessel (v), scale bar = 50 μm. **B**. Representative confocal image of reactive microglia in the peri-infarct region, indicated by arrows **B**., scale bar = 500 μm. Red box indicates region of the peri-infarct within the dorsal cortex used for quantifying numbers of Iba-1 positive cells. Inset depicts threshold-processed image for the same region. **C**. Mean ± SEM number of Iba1 positive cells in dorsal cortex as a function of sex and Panx1, 4 days after permanent MCA occlusion. ***P < 0.001 *vs* wildtype female; *n* = 6 animals per group.

Resting microglia showed relatively smaller cell bodies with several thin processes, whereas activated microglia displayed amoeboid shapes with highly branched short processes [20], in addition to greater Ionized calcium-binding adaptor molecule 1 (Iba1) immuno-reactive labeling within the peri-infarct region (Figure 2A and 2B). Qualitative analysis revealed no morphological differences in Iba1 labeling as a function of Panx1 deletion or between male and female wild type controls. For Iba1 immune-reactive cell counts, two-way analysis indicated significant main effect of Panx1 genotype [F (1, 19) = 5.045; *P* = 0.036] and no significant main effect of sex [F (1, 19) = 3.197; *P* = 0.0897], and significant interaction between sex and genotype [F (1, 19) = 12.72; *P* = 0.002]. Confirmed by post-hoc analysis, the effect of Panx1 deletion to decrease numbers of Iba1 positive cells was observed in female (*P* = 0.0005), but not in male (*P* > 0.5) subjects (Figure 2C).

**Figure 3:**
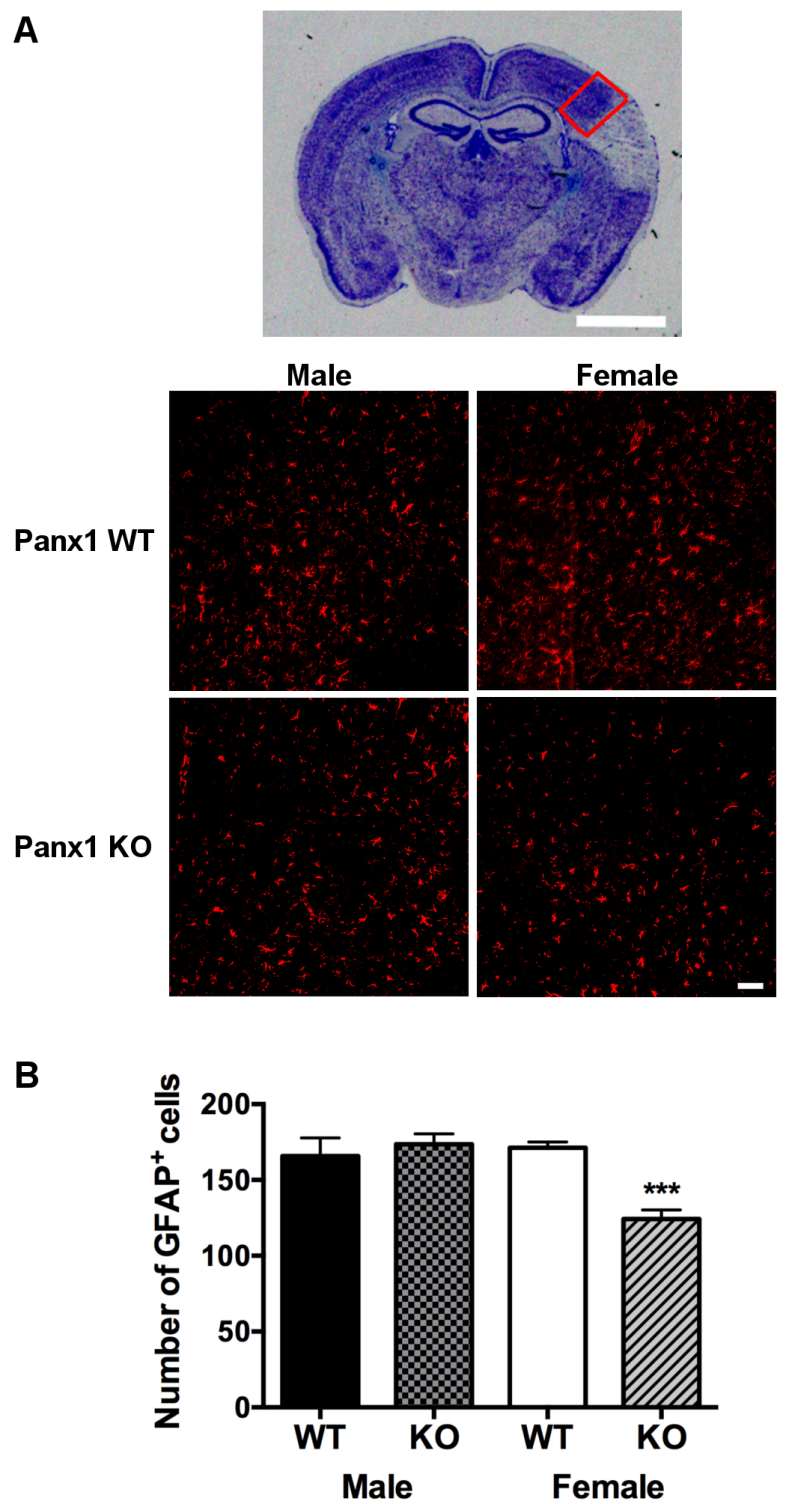
Astrocyte reactivity is reduced in female, but not in male Panx1 KO mice **A**. Representative photomicrograph to show peri-infarct region within the dorsal cortex (red box), used for quantification of GFAP^+^ cells, scale bar = 2 mm. Bottom panels depict higher magnification views of this peri-infarct region, representative of GFAP stained astrocytes (red) from WT and Panx1 KO males and females, 4 days after permanent MCA occlusion, scale bar = 50 μm. **B**. Mean ± SEM number of GFAP^+^ cells in dorsal cortex as a function of sex and Panx1. ***P < 0.001 *vs* wild type females; *n* = 6 animals per group.

Qualitatively, GFAP staining within the peri-infarct region was similar between males and females of either genotype (Figure 3A). With respect to GFAP-immuno-reactivity cell counts, two-way analysis indicated significant main effects of Panx1 genotype [F (1, 21) = 6.405; *P* = 0.0194] and sex [F (1, 21) = 7.968; *P* = 0.0102], and a significant interaction between sex and genotype [F (1, 21) = 12.61; *P* = 0.0019]. Confirmed by post-hoc analysis, the effect of Panx1 deletion to decrease numbers of GFAP positive cells was observed in female (*P* = 0.0004), but not in male (*P* = 0.5) subjects (Figure 3A and 3B).

### Panx1 protein levels

**Figure 4:**
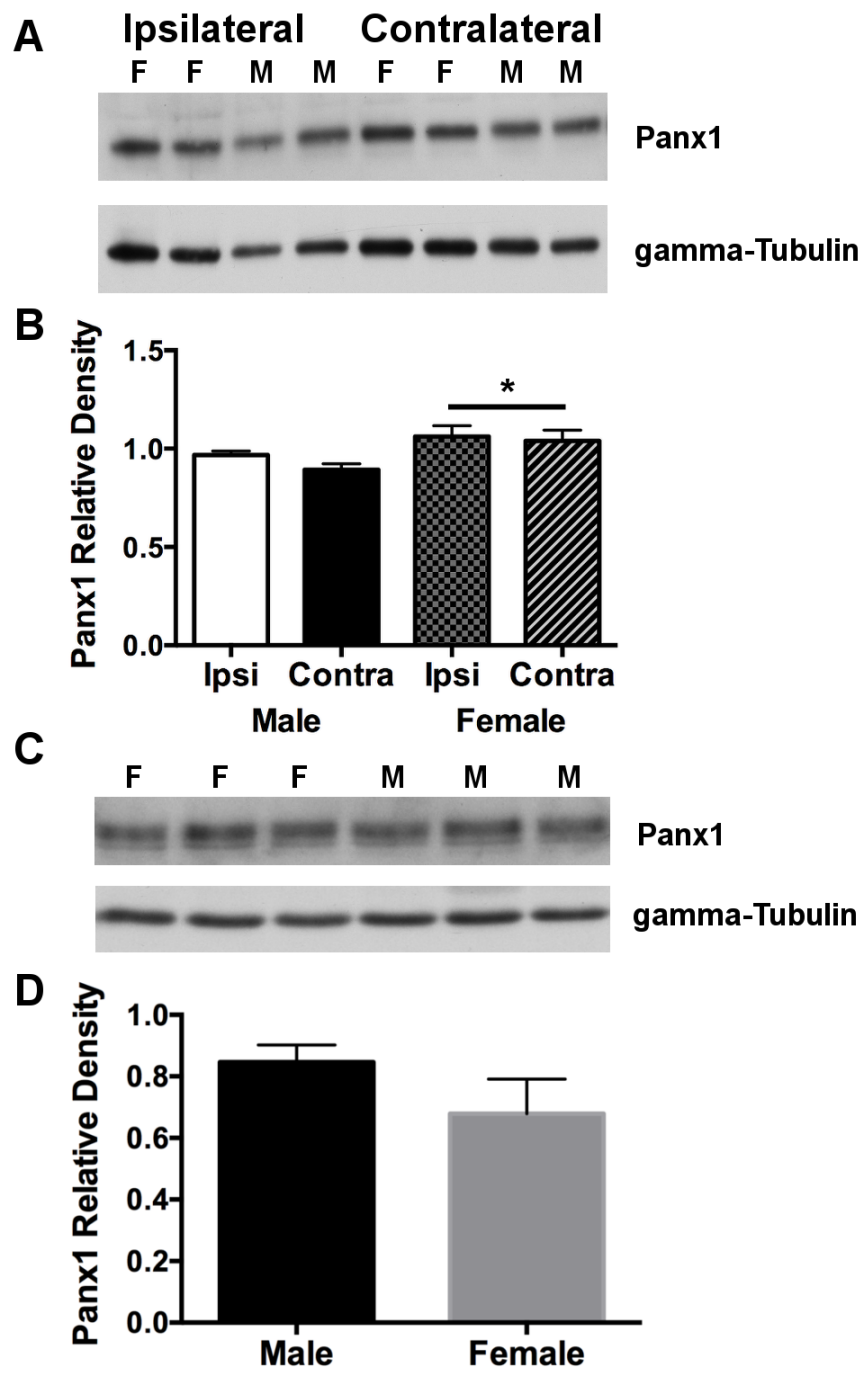
Panx1 protein in cortex is overall higher in females compared to males subjected to permanent MCA occlusion **A**. Western blot of Panx1 protein on sides of cortex ipsilateral (ischemic) and contralateral (non-ischemic) to the MCA occlusion. **B**. Mean ± SEM relative band densities for Panx 1 protein as a function of side of cortex and sex, 4 days after permanent MCA occlusion. *P < 0.05 *vs* males; *n* = 4 animals per group .**C**. Representative western blot of Panx1 protein expression from cortical brain tissue isolated from physiologically normal non-ischemic male and female WT mice. **D**. Mean ± SEM relative band densities for Panx 1 protein expression in male and female cortical tissue. *n* = 5 animals per group. F = female and M = male.

To investigate effects of ischemia and/or sex to alter Panx1 expression in wild type animals, extracts obtained from the sides of cortex ipsi- and contralateral to the MCA occlusion were compared within and between animals. Two-way analysis indicated no significant effect of side ( [F(1, 6) = 0.95; *P* = 0.369], and no interaction between sex and side [F(1, 6) = 0.21; *P* > 0.5]. However, there was a significant effect of sex [F(1, 6) = 7.11; *P* = 0.037], attributed to overall higher Panx1 protein levels in females compared to males (Figure 4A and 4B). Cortical/brain extracts from KO mice rendered undetectable levels of the Panx1 protein (data not shown).

To determine whether Panx1 expression is higher in female mice under normal physiological non-ischemic conditions, cortical brain extracts were isolated from both WT male and female mice and compared between animals. Unpaired *t*-test analysis indicated no significant difference in Panx1 protein expression between male and female WT mice (Figure 4C and 4D).

## DISCUSSION

Present findings indicate that Panx1 ablation and blockade results in a marked reduction in infarct volume, inflammation and gliosis in female, but not in male mice. Panx1 KO female mice exhibited smaller infarct volumes than wild type controls, in addition to reduced microglia and astrocytic reactivity 4 days after permanent MCA occlusion. Pharmacological blockade of Panx1 also produced a similar reduction in infarct volume in wild type females, but not in males. Taken together, these findings unmask important sex differences in Panx1-mediated, inflammatory and neuronal responses to cerebral ischemia.

Panx1 ablation and blockade in male mice have been previously shown to decrease inflammation and neuronal death in response to transient MCA occlusion [12, 14]. This discrepancy with our current findings may be model dependent, as here we employed a distal, permanent model of MCA occlusion. Consistent with our results, Bargiotas [11] showed no significant difference in infarct volume in their Panx1 KO male mice, compared to wild type controls, using a similar permanent model of MCA occlusion. Moreover, unlike previous Panx1 studies employing peripubertal mice (2-3 months), the animals used in the current study were middle aged (8-9 months). These two groups are distinguished by age-dependent shifts in the neuroprotective capacities of testosterone in males and estrogen in females [21–23]. Thus, testosterone in males and estrogen in females appear more and less protective, respectively, in middle aged compared to younger aged animals. In this context, we predict the neuroprotective effects of the gonadal steroids in stroke to be co-opted by age-dependent changes in Panx1 expression and function. For example, a recent study investigating the role of Panx1 in mammary gland development during lactation suggested that Panx1 is downstream of estrogen receptor β (ERβ) signaling pathway. The authors propose that Panx1 ablation may impair ERβ signaling in their model [24]. In addition, de Rivero Vaccari et al. [10] reported that activation of ERβ by exogenous estrogen regulates inflammasome activation and protects the brain from global ischemic damage in reproductively senescent female rats [10]. Interestingly, Panx1 activity is associated with triggering activation of the inflammasome complex [5] and may be repressed by activated ERβ in younger female mice. In wild type animals under ischemic conditions, Panx1 protein levels in cortex were overall higher in females compared to males. In contrast, under non-ischemic conditions, Panx1 protein levels were similar between WT male and female mice. The nature by which females show greater expression and/or ischemic-induced activation of Panx1 remains to be seen, but nonetheless remains consistent with their unique responses to Panx1 deletion and blockade.

Sex differences in stroke-induced signaling cascades [19, 25] provide an important context for understanding why males and females possess different endogenous requirements for Panx1 to alter stroke severity. Ischemia-induced cell death in males is triggered by Poly(ADP-ribose) polymerase (PARP) activation and nuclear translocation of apoptosis-inducing factor (AIF), whereas caspase activation dominates in females [25]. In response to MCA occlusion, female mice exhibit early release of cytochrome-c and enhanced caspase activation [19], where selective pan-caspase inhibition is neuroprotective [19, 25]. Different lines of evidence suggest that Panx1-mediated ATP and UTP release is induced by caspases in apoptotic cells [6, 9, 26, 27], which could serve to further enhance the pro-inflammatory function of reactive microglia and triggering of neuronal death [28]. In this design, the predominant caspase-dependent cell death pathway in females could provide a mechanism for greater ATP release and Panx1 activation, consistent with their unique responses to Panx1 blockade and knockout, in addition to reduced levels of peri-infarct astrocytic and microglia reactivity.

We have only just begun to understand sex and Panx1 interactions in stroke, as no study reported to date has incorporated females into studies of Panx1 ablation and permanent MCA occlusion. The findings presented here highlight Panx1 as a potential important player associated with sex differences in stroke.

The specific mechanism(s) through which Panx1 affects stroke in females is unclear. We have highlighted above two potential mechanisms, that is, through the predominant caspase-dependent cell death pathway in females or the ERβ mediated regulation of the inflammasome, with which Panx1 is associated. Whether either of these two pathways affects Panx1 activity in females, subjected to stroke, is an important question to be addressed in future studies.

Our findings with Panx1 knockout mice are supported in additional experiments where we use probenecid as a Panx1 blocker. There is a growing body of evidence showing the effects of probenecid on Panx1 activity [8, 9, 29–32]*.* More importantly, the fact that probenecid is already used clinically makes this drug an ideal candidate for therapeutic treatments involving Panx1. This is the primary reasoning for the selection and use of this drug in this study.

In conclusion, despite the strength to which Panx1 operates on the stroke response in various experimental models, and the growing list of agents targeting this pannexin (and other cell membrane channels), the bulk of our current understanding of stroke and Panx1 interactions remains seated in studies employing male subjects only. Nonetheless, our current findings make clear a major sex difference in neuroprotection afforded by Panx1 ablation that occurred in females, but not in males. Thus, Panx1 blockade has realistic clinical applications, both as a therapeutic agent and as an adjunct to existing sex hormone replacement regimens, and promises to lead to more appropriate treatment strategies for men and women. These findings suggest that the endogenous requirements for Panx1 to regulate neuronal responses to ischemic injury are seemingly different between males and females, and provide several new frameworks for understanding how Panx1 may come to link sex-dependent variations in stroke outcome responses.

## MATERIALS AND METHODS

### Animals and permanent middle cerebral artery (MCA) occlusion

Panx1 WT and Panx1 KO C57BL/6 mice [6], kindly provided by Dr. Dale W. Laird (University of Western Ontario, Canada), were maintained on a 12:12 h light: dark cycle, with food and water available *ad libitum*. For Panx1 blockade, probenecid (Sigma-Aldrich, Canada) was dissolved in 0.5 N NaOH and adjusted to pH 7.4 with saturated KH_2_PO_4_, as previously described [33]. WT male (*N* = 6) and female (*N* = 6) mice received two injections of probenecid, one (250 mg/kg i.p) at 1.5 hours after MCA occlusion and a second injection (250 mg/kg i.p.) 5.5 hours after MCA occlusion [27]. The control cohort received sterilized vehicle injections. The time-points chosen for probenecid administration in this study were based on the clinical and research data that neuroprotective agents must be administered within 5 hours after stroke onset [34]; this is consistent with the tissue plasminogen activator (tPA) administration protocol [35]*.* All breeding and animal procedures were approved by The University of British Columbia Animal Care Committee.

The average weight of WT mice (male *N* = 7 and female *N* = 9) for males was 36.46 ± 1.312 g and for females was 28.13 ± 1.696 g. For Panx1 KO mice (male *N* = 9 and female *N* = 9) the average weight for males was 33.37 ± 0.5525 g and for females was 25.17 ± 1.073 g. These mice were subjected to permanent MCA occlusion, as previously described [36, 37]. All surgical procedures were performed under sterile conditions. Briefly, 8 to 9 month-old mice were anesthetized with sodium pentobarbital (65 mg/kg i.p.). The head was held securely in place using a stereotaxic frame (David Kopf Instruments, USA). With the aid of a dissecting microscope (Hund Wetzlar, Germany), a skin incision was made on the right side of the head from the anterior of the ear towards the corner of the eye horizontally and from the corner of the eye vertically 5 mm. The squamosal bone was exposed by gently pulling back the temporal muscle. Using a fine battery-powered drill (Dremel, Canada), a small hole was made ~2 mm in diameter on the skull bone to remove dura and expose the MCA. The MCA was then cauterized above and below the rhinal fissure using an electronic coagulator (Codman & Shurtleff Inc., USA). After cauterizing the MCA absence of reperfusion was visually confirmed 10 min after occlusion, the skin incision was then closed with sutures and mice were given a 1 ml subcutaneous bolus of lactate Ringer's solution kept at 37 °C. During surgery, mice were maintained at 37 °C on a heating pad, the temperature in the operating room was maintained at 24 °C. Measurements were obtained *via* rectal temperature only during surgery. Brain temperature was not monitored. Immediately after surgery mice were placed in a recovery cage on a heating pad set at 37 °C. When mice were fully recovered from anesthesia (~ 2 hr), the animals were then placed in their home cages on a heating pad set at 37 °C for 12 hr. Mice were then transferred to a temperature controlled (22.0 °C ) recovery room for the remaining 4 days and monitored daily. Hyperthermia or hypothermia were not assessed in mice after surgery [38].

### Quantification of cerebral infarction

Infarct volume was quantified as previously described [39]. Four days after permanent MCA occlusion, WT and Panx1 KO mice were anesthetized using a lethal dose of sodium pentobarbital (100 mg/kg i.p.) and transcardially perfused with phosphate-buffered saline (PBS), followed by 10% formalin (Sigma-Aldrich, Canada). The brains from each mouse were dissected out from the skull and immediately placed in 10% formalin for 8 hours at 4 °C. The brains were then washed in PBS and transferred into tubes filled with 30% sucrose in PBS, and were left in this solution at 4 °C until further proccessing. A cryostat (HM 505E; Micron, Walldorf, Germany) was used to obtain 20 μm- and 10 μm-thick brain sections, collected at 100 µm intervals for infarct volume determination and immunohistochemistry. To measure infarct size, sections were stained with 0.125% thionin (Fisher Scientific, Canada). Total infarct volumes were calculated using a stereological approach through the rostrocaudal extent of the infarct area, and corrected for edema as previously described [40].

### Western blot analysis

In order to assess the level of Panx1 protein, additional cohorts of WT (male *N* = 4 and female *N* = 4) and Panx1 KO (male *N* = 4 and female *N* = 4) mice were likewise euthanized with sodium pentobarbital (100 mg/kg i.p.) 4 days after permanent MCA occlusion and perfused with ice-cold PBS. A total of 8 serial, 20 µm-thick sections were used to isolate protein from both ipsilateral (ischemic) and contralateral (non-ischemic) sides of cortex from individual animals [41]. To determine basal levels of Panx1 expression in normal non-ischemic brain, a cohort of 9-month-old animals (male *N* = 5 and female *N* = 5) were used and cortical brain tissue was dissected for protein isolation. Protein samples were isolated using RIPA buffer and quantified using the BCA method (Pierce, USA), resolved by sodium dodecyl sulfate-polyacrylamide gel electrophoresis [42]. Briefly, 40 μg of protein was separated on a 10% sodium dodecyl sulfate-polyacrylamide gel and transferred to polyvinylidene difluoride membranes. Membranes were processed and incubated overnight at 4°C with primary antibodies against rabbit anti-Panx1 (provided by Dr. Dale W. Laird), or mouse anti-γ-tubulin (catalog number: T6557; Sigma-Aldrich, Canada), in TBST containing 1% skimmed milk. The membranes were washed and incubated with horseradish peroxidase-conjugated secondary antibody (Sigma-Aldrich, Canada) 1/5000 in TBST containing 5% skimmed milk. Immunoreactive proteins were visualized by chemiluminescent solution (Super Signal West Pico, Pierce Biotechnology Inc, USA). Densitometric, semi-quantitative analysis of western blots was performed using ImageJ software [43]. Band intensity measurements obtained from proteins of interest were normalized to housekeeping protein (γ-tubulin) values. In addition, individual western blots were loaded with a standard sample obtained from a mixture of E18 and 9-month old, ischemic brain homogenates, to permit comparisons made between blots.

### Immunofluorescent histochemical detection of neuroinflammatory responses

Inflammation was assessed 4 days following MCA occlusion; we have extensively studied the time course of injury following MCA occlusion, and 4 days provides an optimal time course to assess not only infarct volume but also neuronal injury and glial reactivity [37]. Immunofluorescence was performed as previously described [42]. Incubation of primary antibodies were performed overnight at 4°C, in PBS + 10 % goat serum. Primary antibodies and their dilutions used were as follows: rabbit anti-ionized calcium-binding adaptor molecule 1 (Iba1) (1:400; catalog number: 019-19741; Wako, USA); mouse anti-GFAP (1:1000; catalog number: G3893; Sigma-Aldrich, Canada). Appropriate secondary Alexafluor^®^ antibodies (Invitrogen, Canada) were used at 1:500 dilutions in PBS buffer. Sections were mounted with Prolong Gold antifade reagent with DAPI (Invitrogen, Canada). Omission of primary antibodies served as negative controls.

Histological quantification was performed as previously described [42]. Male and female WT (*N* = 6 / group) and Panx1 KO (*N* = 6 / group) were analyzed under control and ischemic conditions. Three serial brain sections (100 µm intervals) from each animal at the level of the frontal cortex were used for immunofluorescent analysis. Images containing the peri-ischemic area, specifically in the dorsal cortex, were captured by confocal microscopy (Leica Nussloch, Germany) using a Zeiss Axioplan2 fluorescence microscope (Carl Zeiss Ltd, Canada). For quantification of GFAP^+^ and Iba1^+^ cell numbers, several selected field (420 × 820 µm) encompassing the peri-infarct region of the dorsal cortex were analyzed using ImageJ software.

### Statistics

Single two-way ANOVAs (between sex and treatment) were used to explore most variables of interest. A two-way mixed design ANOVA (between sex, within subject) was used for Western analysis using side of cortex (ipsi- and contralateral) as repeated measure. When appropriate, post-hoc comparisons were made using the Tukey-Kramer test for unequal sample sizes. Unpaired *t* tests were performed when comparing between two groups.

## Abbreviations

AIF: Apoptosis-inducing factor
ATP: Adenosine triphosphate
ERβ: Estrogen receptor β
GFAP: Glial fibrillary acidic protein
Iba1: Ionized calcium-binding adaptor molecule 1
KO: Knockout
MCA: middle cerebral artery
OGD: Oxygen and glucose deprivation
Panx1: Pannexin 1
PARP: Poly(ADP-ribose) polymerase
PBS: Phosphate-buffered saline
WT: Wild type.
